# Differences in the photosynthetic and physiological responses of *Leymus chinensis* to different levels of grazing intensity

**DOI:** 10.1186/s12870-019-2184-1

**Published:** 2019-12-16

**Authors:** Min Liu, Jirui Gong, Bo Yang, Yong Ding, Zihe Zhang, Biao Wang, Chenchen Zhu, Xiangyang Hou

**Affiliations:** 1 Beijing Key Laboratory of Traditional Chinese Medicine Protection and Utilization, Key Laboratory of Surface Processes and Resource Ecology, College of Resources Science and Technology, Faculty of Geographical Science, Beijing Normal University, No. 19 Xinjiekouwai Street, Beijing, 100875 China; 2 Key Laboratory of Tourism and Resources, Environment in Taishan University, Taian, 271021 China; 3 Grassland Research Institute of Chinese Academic of Agricultural Science, Hohhot, 010021 Inner Mongolia China

**Keywords:** Grazing intensity, Photosynthetic capacity, Chlorophyll fluorescence, Chloroplast structure, Reactive oxygen species

## Abstract

**Background:**

Grazing is an important land use in northern China. In general, different grazing intensities had a different impact on the morphological and physiological traits of plants, and especially their photosynthetic capacity. We investigated the responses of *Leymus chinensis* to light, medium, and heavy grazing intensities in comparison with a grazing exclusion control.

**Results:**

With light grazing, *L. chinensis* showed decreased photosynthetic capacity. The low chlorophyll and carotenoid contents constrained light energy transformation and dissipation, and Rubisco activity was also low, restricting the carboxylation efficiency. In addition, the damaged photosynthetic apparatus accumulated reactive oxygen species (ROS). With medium grazing, more energy was used for thermal dissipation, with high carotene content and high non-photochemical quenching, whereas photosynthetic electron transport was lowest. Significantly decreased photosynthesis decreased leaf C contents. Plants decreased the risk caused by ROS through increased energy dissipation. With high grazing intensity, plants changed their strategy to improve survival through photosynthetic compensation. More energy was allocated to photosynthetic electron transport. Though heavy grazing damaged the chloroplast ultrastructure, adjustment of internal mechanisms increased compensatory photosynthesis, and an increased tiller number facilitated regrowth after grazing.

**Conclusions:**

Overall, the plants adopted different strategies by adjusting their metabolism and growth in response to their changing environment.

## Background

Grazing is the most common and important land use in Inner Mongolia that affects grassland productivity and vegetation dynamics [67, 83]. In recent years, more and more grasslands have been severely damaged by long-term overgrazing, resulting in widespread grassland degradation [43, 84]. The over-grazing decreases vegetation cover and damages the soil, leading to desertification and continuously decreasing grassland productivity, thereby damaging the structure and functions of the grassland ecosystem [6]. The dominant grassland species contribute most to the ecosystem’s productivity and therefore play an important role in conferring resistance to disturbance and maintenance of stability. Therefore, it’s essential that we improve our understanding of how the dominant species respond to the grazing disturbance.

Livestock grazing directly affects plant morphology. For example, it reduces plant height, decreases the length of shoot internodes, and decreases leaf area [76, 88]. Because taller plants with more leaves are more attractive to herbivores, the dwarf characteristics that result from grazing may help the plants to escape herbivores [77]. At the same time, grazed plants increase their tiller production to promote rapid regrowth [87]. On the other hand, grazing stress also affected the physiological responses of plants, such as compensatory growth and changes in photosynthetic capacity [12, 49, 63].

Herbivory was formerly thought to be detrimental to plant fitness because of its metabolic effects, and particularly down-regulation of photosynthesis [26]. However, the primary response to removal of tissues by herbivory is regrowth through the reconstruction of damaged tissues and organs, which is achieved by increased CO_2_ assimilation capacity. The plant responds by increasing the chlorophyll content, activity of photosynthetic enzymes, and electron transport capacity, which together improves the physiological functioning of the photosynthetic apparatus. These changes may be sufficient to compensate for the loss of photosynthetically active leaves [30, 34]. Furthermore, grazing removes old and dead plant tissues and alters the plant’s mass allocation so that plants can produce more new leaves to restore their photosynthetic capacity [87, 88].

As these responses show, plants that face different grazing stress may exhibit different responses and different protection strategies. Because damage to plants increases with increasing grazing intensity, plant photosynthetic properties are also likely to change. However, we still don’t fully understand the physiological changes that occur under different grazing intensities.

In general, the absorption and utilization of solar energy by plants changes in response to grazing. Plants have more light available to them after grazing due to the decreased shading, but on the other hand, the reduced leaf area may limit their ability to acquire sufficient light [70]. Increased illumination may also cause photoinhibition and decreased photosynthetic efficiency if the input of photons exceeds the plant’s photosynthetic capacity [28, 46, 63]. When the excess energy cannot be safely dissipated, this leads to the accumulation of reactive oxygen species (ROS) [75, 85, 89]. The ROS suppress the synthesis of PSII proteins in the chloroplasts, increase lipid peroxidation, and damage the photosynthetic apparatus [13, 71].

CO_2_ assimilation by the chloroplasts consumes energy and depends on Rubisco (ribulose 1,5-bisphosphate carboxylase) [46]. Thus, any stress that adversely affects Rubisco activity or regeneration will reduce photosynthesis. Grazing and other environmental stresses can also accelerate the degradation of chloroplasts, resulting in swollen or ruptured chloroplasts in the damaged leaves [20, 66]. Overall, the available evidence suggests that the plant’s photosynthetic capacity is affected by the structural and the physiological responses to grazing [29, 68, 81]. But little information is available on the structural changes of chloroplasts and the associated regulatory mechanisms under grazing.

Plants develop many strategies to scavenge ROS and protect their photosynthetic apparatus against stress-induced damage. For example, the presence of large quantities of xanthophyll-cycle components in leaves can consume excess energy and provide protection against excessive light energy, thereby reducing the production of ROS [53]. At the same time, the plant’s system of antioxidant enzymes, which include superoxide dismutase (SOD), catalase (CAT), ascorbate peroxidase, glutathione peroxidase, and peroxidase (POD), plays a crucial role in scavenging ROS [4, 11]. In addition, up-regulation of proline production can mitigate the reduction in peroxidase and catalase activity that sometimes occurs under stress [33]. Proline can decrease ROS levels and delay or prevent cell death [10]. In addition, proline accumulation makes the cell osmotic potential more negative, thereby increasing resistance to the water stress that results from grazing damage [27]. Although many studies have illustrated the roles of these antioxidant enzymes, the relationship between the stress and enzyme activity is not clear. The specific strategies that plants adopt to cope with stress, and the relationship of these strategies to the plant’s grazing tolerance, are still mostly unknown and need to be discovered.

Grasslands cover almost 42% of the earth’s surface and account for about 34% of the global terrestrial organic carbon storage, which makes them a widespread and important vegetation type [68]. In China, grassland covers more 40% of the total land area [37]. As the main temperate grassland of northern China, Inner Mongolia’s grasslands play a crucial role in environmental protection and livestock production [86]. However, the grasslands are fragile and sensitive to human disturbance, and especially to irrational utilization and unsustainable development [42, 82]. To provide some of the missing knowledge of grazing effect on grassland, we designed the present study to examine the effects of four different grazing intensities (a control with grazing exclusion, and light, medium, and heavy grazing) on the photosynthetic capacity of *Leymus chinensis*, a key species in northern China’s grassland ecosystems. *L. chinensis* is the widely distributed grass in Inner Mongolia’s grasslands. It is a perennial C3 grass, with the long and strong rhizomes. *L. chinensis* shows a vigorous vegetative propagation in the grassland [45]. To do so, we measured gas exchange, chlorophyll fluorescence, photosynthetic enzyme activity, pigment contents, and the ultrastructure of the chloroplasts. We also measured the peroxidation of membrane lipids and the plant’s antioxidant system. Our specific objectives were (i) to clarify the photosynthetic response and adaption mechanisms of *L. chinensis* at different grazing intensities; (ii) to examine the relationship between the structural changes of chloroplasts and physiological regulation of photosynthesis under the different grazing intensities; and (iii) to identify the equilibrium between the generation and elimination of ROS.

## Results

### Plant morphological traits and soil traits

The morphological characteristics of *L. chinensis* differed significantly among the four grazing plots (Table 1, *P* < 0.05). SLA was highest in the LG plots and lowest in the MG plots. The tiller number increased with increasing grazing intensity, and the difference was significant in the HG plots, whereas the mean internode length decreased gradually, and the difference was significant for all grazing intensities. Plant biomass and height decreased with increasing grazing intensity, and the differences became significant in the HG and MG plots, respectively. Especially in the HG plots, the biomass and height reduced by half. Leaf osmotic potential decreased (became more negative, indicating greater water stress) with increasing grazing intensity, and the difference was significant in the MG and HG plots.

**Table 1:** The morphological traits of *L. chinensis* and the soil characteristics in the plots with different grazing intensities

Parameter	Control	LG	MG	HG
Specific leaf area (cm^2^ g^− 1^)	116.1 ± 2.6b	143.4 ± 3.7a	100.6 ± 4.1c	118.8 ± 6.9b
Tiller number	3.4 ± 0.5b	4.0 ± 0.4ab	4.2 ± 0.4ab	5.3 ± 0.3a
Mean internode length (cm)	6.1 ± 0.1a	5.4 ± 0.3b	2.4 ± 0.2c	2.4 ± 0.2c
Plant height (cm)	26.3 ± 0.8a	24.6 ± 1.1a	20.4 ± 0.7a	13.2 ± 1.6b
Plant biomass (g m^−2^)	35.1 ± 1.7a	31.3 ± 2.0ab	27.6 ± 2.4b	15.2 ± 1.0c
Leaf osmotic potential (MPa)	−1.6 ± 0.04a	−1.9 ± 0.19ab	−3.0 ± 0.04b	−2.8 ± 0.92b
Soil water content (%)	5.77 ± 0.93c	7.60 ± 0.08b	8.95 ± 0.11a	4.53 ± 0.08d
Soil nitrogen content (%)	0.15 ± 0.003b	0.17 ± 0.003a	0.16 ± 0.001a	0.14 ± 0.003c
Soil carbon content (%)	1.47 ± 0.018c	1.64 ± 0.005a	1.54 ± 0.015b	1.37 ± 0.006d
Soil available phosphorus (%)	405.6 ± 0.66a	389.0 ± 6.22b	390.2 ± 3.29b	358.8 ± 1.57c

Values are means ± SE. Values in a column labeled with different letters differ significantly (ANOVA followed by LSD test, *P* < 0.05)

*Control* no grazing, *LG* light grazing, *MG* medium grazing, *HG* heavy grazing

The characteristics of soil were significantly different among the four grazing plots (Table 1, *P* < 0.05). Compare with soil in the control plots, soil in the LG and MG plots was moister, and driest in the HG plots. Similar with the soil water content, the soil nitrogen and carbon contents were higher in the LG and MG plots than those in the control plots, and lowest in the HG plots. In contrast, the soil available phosphorus mainly decreased as the grazing intensity increased.

### The leaf C, N, and P contents

**Fig. 1:**
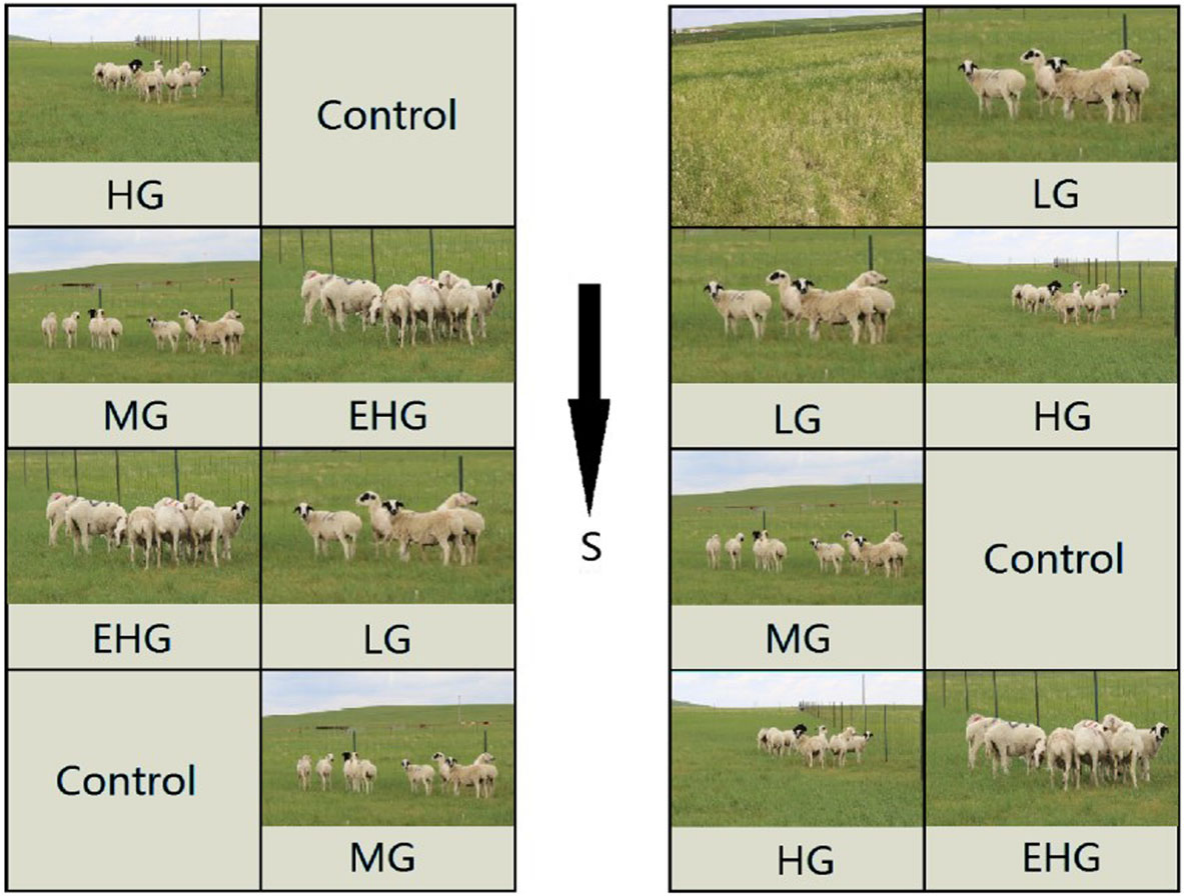
The experimental design of grazing intensity at the study sites (control, no grazing; LG, light grazing; MG, medium grazing; HG, heavy grazing)

The leaf C, N and P contents showed different trends with increasing grazing intensity (Fig. 1). The leaf C content was significantly lower in the MG plots than in the other plots, which did not differ significantly from each other. Leaf N content was significantly higher in the HG plots, but there was no significant difference among the other three plots. In contrast, the P content was significantly higher than in the control in the LG plots, and significantly lower than in the control and in the MG and HG plots.

### The photosynthetic pigment contents and Rubisco activity

**Fig. 2:**
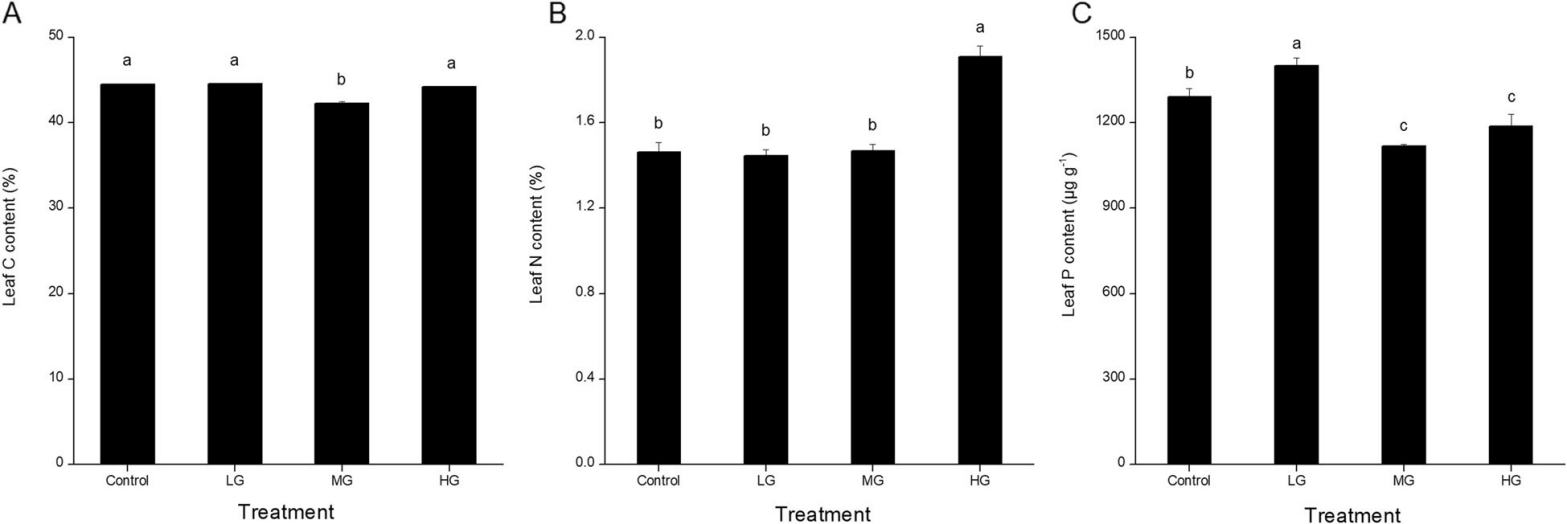
The leaf C, N, and P contents of *L. chinensis* leaves in the plots with different grazing intensities (control, no grazing; LG, light grazing; MG, medium grazing; HG, heavy grazing). Values are means ± SE. Bars labeled with different letters differ significantly (ANOVA followed by LSD test, *P* < 0.05)

Figure 2 shows the leaf contents of the photosynthetic pigments. The contents differed significantly among the grazing intensities (*P* < 0.05). The Chl *a*, Car, and Chl *a* + *b* contents were significantly lower than in the control in the LG plots, but the LG values were significantly lower than in the MG and HG plots. In contrast, the Chl *b* content and the Chl *a*/*b* ratio did not differ significantly among the grazing intensities. The Rubisco activity increased with increasing grazing intensity, but was significantly lower than in the control and in the LG plots and significantly higher in the HG plots.

### Leaf gas-exchange and chlorophyll fluorescence parameters

Table 2 summarized the leaf gas-exchange and response curves characteristics. These differed significantly among the grazing intensities (*P* < 0.05). In the control plots, *L. chinensis* had significantly higher *P*_n_ and WUE than in the grazed plots, but significantly lower *E*. In the LG plots, LSP, and AQY were significantly higher than in the other plots and LCP was significantly lower. In the MG plots, *P*_n_, and WUE were significantly lower than those in the control. In the HG plots, *E* was significantly higher than in the other grazed plots. *R*_d_ was significantly lower in the LG and HG plots than in the control and MG plots, but did not differ significantly between LG and HG or between the control and MG. Table 2 also summarizes the data from the leaf CO_2_-response curves. All four parameters differed significantly among the grazing intensities (*P* < 0.05). *J*_max_, *V*_cmax_, and *V*_TPU_ were significantly higher in the LG plots than in the other plots, and significantly lower in the control plots. In contrast, the *J*_max_ / *V*_cmax_ ratios were lower in the MG plots and significantly higher in the HG plots.

**Table 2:** The gas-exchange characteristics and the light and CO_2_-response curve parameters of *L. chinensis* in the plots with different grazing intensities

	Control	LG	MG	HG
*P*_n_ (μmol m^−2^ s^−1^)	8.46 ± 0.99a	1.64 ± 0.14bc	0.75 ± 0.34c	3.24 ± 0.76b
*T*_r_ (mmol m^−2^ s^−1^)	0.67 ± 0.09d	1.98 ± 0.24b	1.46 ± 0.12c	2.73 ± 0.01a
WUE (μmol mmol^−1^)	13.65 ± 3.77a	0.83 ± 0.02b	0.51 ± 0.23b	1.18 ± 0.28b
LSP (μmol·m^−2^ s^−1^)	492 ± 1.2b	712 ± 4.5a	501 ± 6.4b	542 ± 2.8b
LCP (μmol·m^−2^ s^−1^)	68 ± 3.2a	28 ± 1.2b	54 ± 4.1a	32 ± 2.0b
*R*_d_ (μmol·m^−2^ s^−1^)	2.50 ± 0.18a	1.37 ± 1.24b	2.84 ± 0.21a	1.22 ± 1.28b
AQY (μmol CO_2_ μmol^−1^)	0.039 ± 0.01b	0.049 ± 0.02a	0.037 ± 0.01b	0.039 ± 0.01b
*J*_max_ (μmol·m^−2^ s^−1^)	22.42 ± 1.32d	48.10 ± 2.40a	27.49 ± 2.34c	31.19 ± 1.36b
*V*_cmax_ (μmol·m^−2^ s^−1^)	21.89 ± 0.24c	44.30 ± 5.42a	27.34 ± 1.86b	28.29 ± 3.42b
*V*_TPU_ (μmol·m^−2^ s^−1^)	5.22 ± 0.23c	10.27 ± 0.89a	6.82 ± 0.58b	6.25 ± 0.56b
*J*_max_/*V*_cmax_	1.024 ± 0.02b	1.085 ± 0.03b	1.005 ± 0.01c	1.102 ± 0.03a

Values are means ± SE (*n* = 3). Values of a parameter labeled with different letters differ significantly between light intensities (ANOVA followed by LSD test, *P* < 0.05)

*Control* no grazing, *LG* light grazing, *MG* medium grazing, *HG* heavy grazing, *P*_n_ net photosynthetic rate, *T*_r_ transpiration rate, *WUE* water-use efficiency, *LSP* light saturation point, *LCP* light compensation point, *R*_*d*_ dark respiration rate, *AQY* apparent quantum yield, *J*_*max*_ maximum electron transport rate, *V*_*cmax*_ maximum carboxylation efficiency, *V*_*TPU*_ triose phosphate utilization rate

**Fig. 3:**
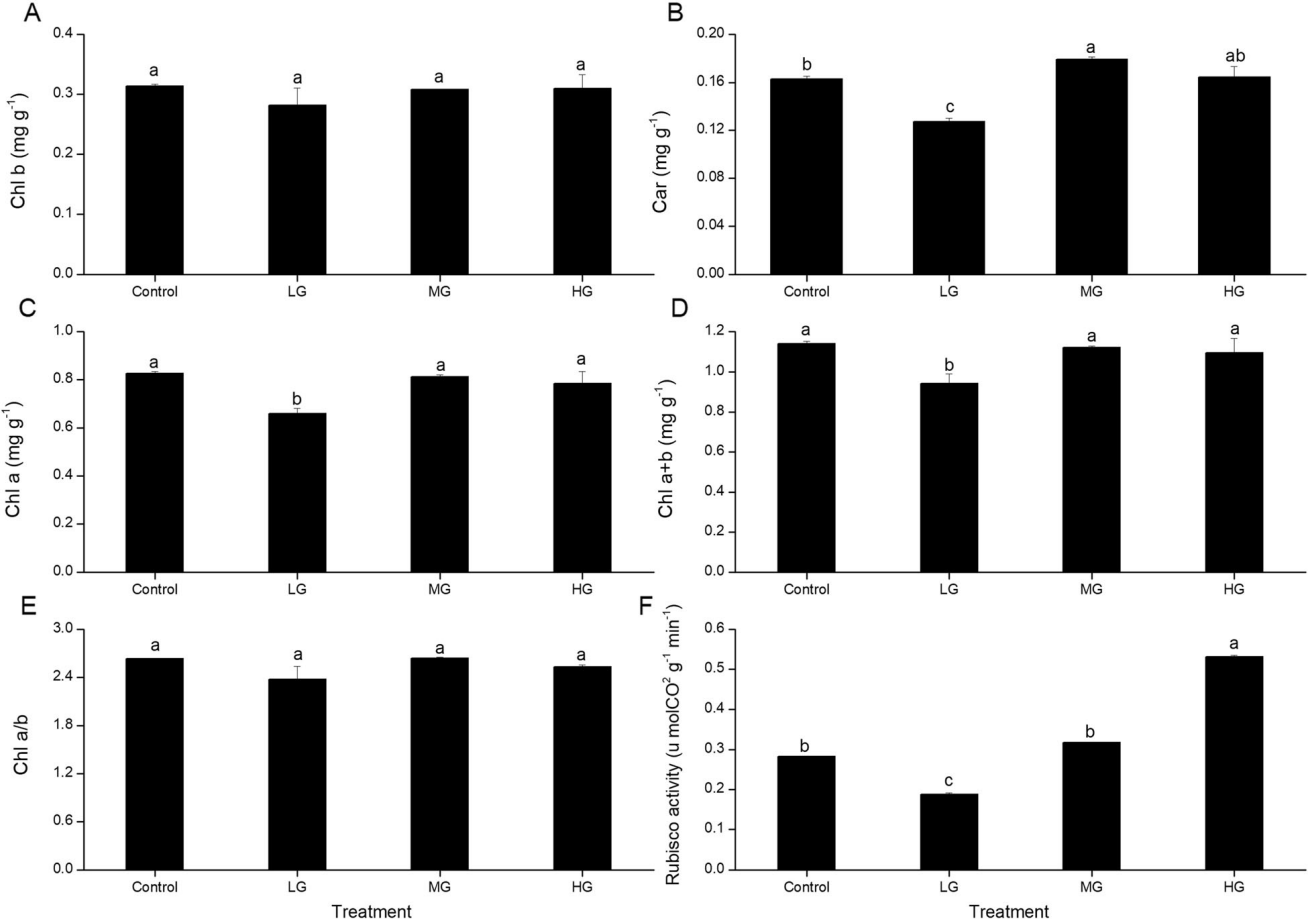
The chlorophyll a (Chl *a*), chlorophyll b (Chl *b*), and carotenoid (Car) contents, total chlorophyll content (Chl *a* + *b*), chlorophyll a/b ratio (Chl *a*/*b*), and Rubisco activity in leaves of *L. chinensis* in the plots with different grazing intensities (control, no grazing; LG, light grazing; MG, medium grazing; HG, heavy grazing). Values are means ± SE (*n* = 3). Values of a parameter labeled with different letters differ significantly between grazing intensities (ANOVA followed by LSD test, *P* < 0.05)

Table 3 summarizes the leaf chlorophyll fluorescence parameters, which differed significantly among the grazing intensities (*P* < 0.05). There was no significant difference in *F*_v_/*F*_m_, qP, and NPQ among the plots. In the control plots, the values of *F*_v_’/*F*_m_′ were significantly higher than those in the other plots. In the LG plots, *F*_m_ and *F*_v_’/*F*_m_′ were significantly lower than in the control. In the MG plots, *F*_0_, *F*_m_, *F*_v_’/*F*_m_′, and Φ_PSII_ were significantly lower than in the other plots. Compared with the MG plots, more energy was used for photosynthetic electron transport in the HG plots and less was used in the MG plots. Thermal dissipation was higher and excess energy was lower than in the control in all of the grazed plots (Fig. 3).

**Table 3:** The chlorophyll fluorescence parameters of *L. chinensis* in the plots with different grazing intensities

	Control	LG	MG	HG
*F*_o_	133.9 ± 6.53c	184.3 ± 5.58a	123.8 ± 6.70c	155.8 ± 2.51b
*F*_m_	646.5 ± 13.0a	624.9 ± 46.1ab	502.2 ± 30.0c	599.3 ± 23.5b
*F*_v_/*F*_m_	0.79 ± 0.005a	0.76 ± 0.006a	0.78 ± 0.001a	0.77 ± 0.005a
*F*_v_ ‘/*F*_m_’	0.501 ± 0.021a	0.424 ± 0.001b	0.377 ± 0.005c	0.446 ± 0.013b
*q*_P_	0.509 ± 0.056a	0.583 ± 0.027a	0.503 ± 0.036a	0.595 ± 0.007a
NPQ	1.305 ± 0.230a	1.328 ± 0.557a	1.545 ± 0.444a	1.289 ± 0.252a
ETR	166.8 ± 16.8ab	130.0 ± 6.24b	150.0 ± 13.0ab	174.4 ± 6.77a
Φ_PSII_	0.254 ± 0.025a	0.247 ± 0.012a	0.190 ± 0.016b	0.266 ± 0.010a

Values are means ± SE (*n* = 3). Values of a parameter labeled with different letters differ significantly between light intensities (ANOVA followed by LSD test, *P* < 0.05)

*Control* no grazing, *LG* light grazing, *MG* medium grazing, *HG* heavy grazing, *F*_0_ minimum fluorescence, *F*_*m*_ maximum fluorescence, *F*_*v*_*/F*_*m*_ the maximum quantum efficiency of PSII, *F*_*v*_*’/F*_*m*_*′* energy harvesting efficiency of PSII, *q*_*P*_ photochemical quenching coefficient, *NPQ* non-photochemical quenching coefficient, *ETR* electron transport rate, *Φ*_*PSII*_ effective quantum yield of PSII

**Fig. 4:**
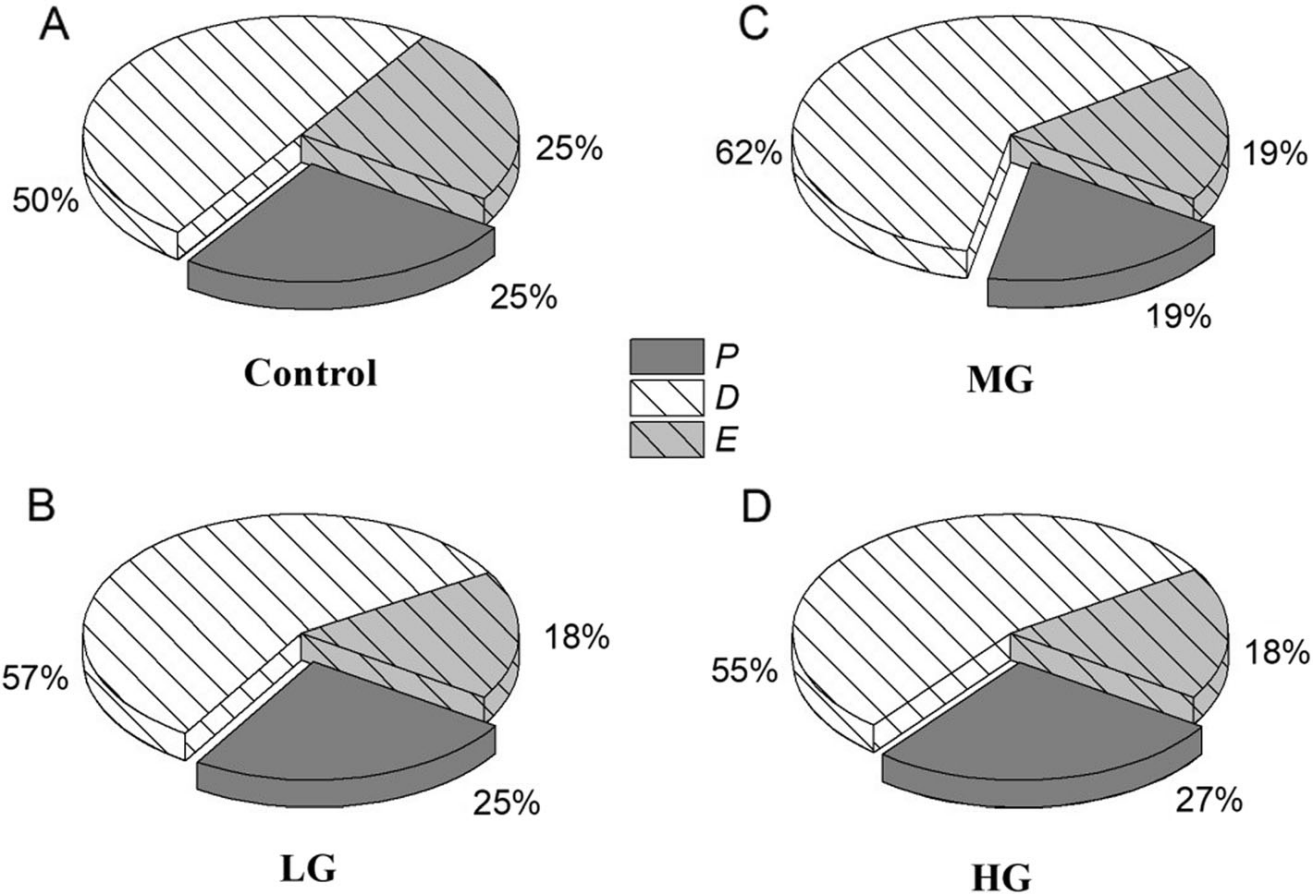
The energy partitioning of *L. chinensis* in the plots with different grazing intensities (control, no grazing; LG, light grazing; MG, medium grazing; HG, heavy grazing). Energy types: *P*, photosynthetic electron transport; *D*, thermal dissipation; *E*, excess energy

In the control plots, the chloroplasts were intact, with an orderly arrangement of grana and of the stroma lamellae and well-developed thylakoid membranes (Fig. 4a). In the LG plots, starch grains were occasionally observed in the chloroplasts (Fig. 4b). There was no obvious difference of chloroplast ultrastructure among the grazing intensity except in the HG plots, where the chloroplasts were swollen and had irregular grana (Fig. 4d). In addition, the degraded osmiophilic granule became common in the chloroplasts in the HG plots.

### Lipid peroxidation and antioxidant systems

**Fig. 5:**
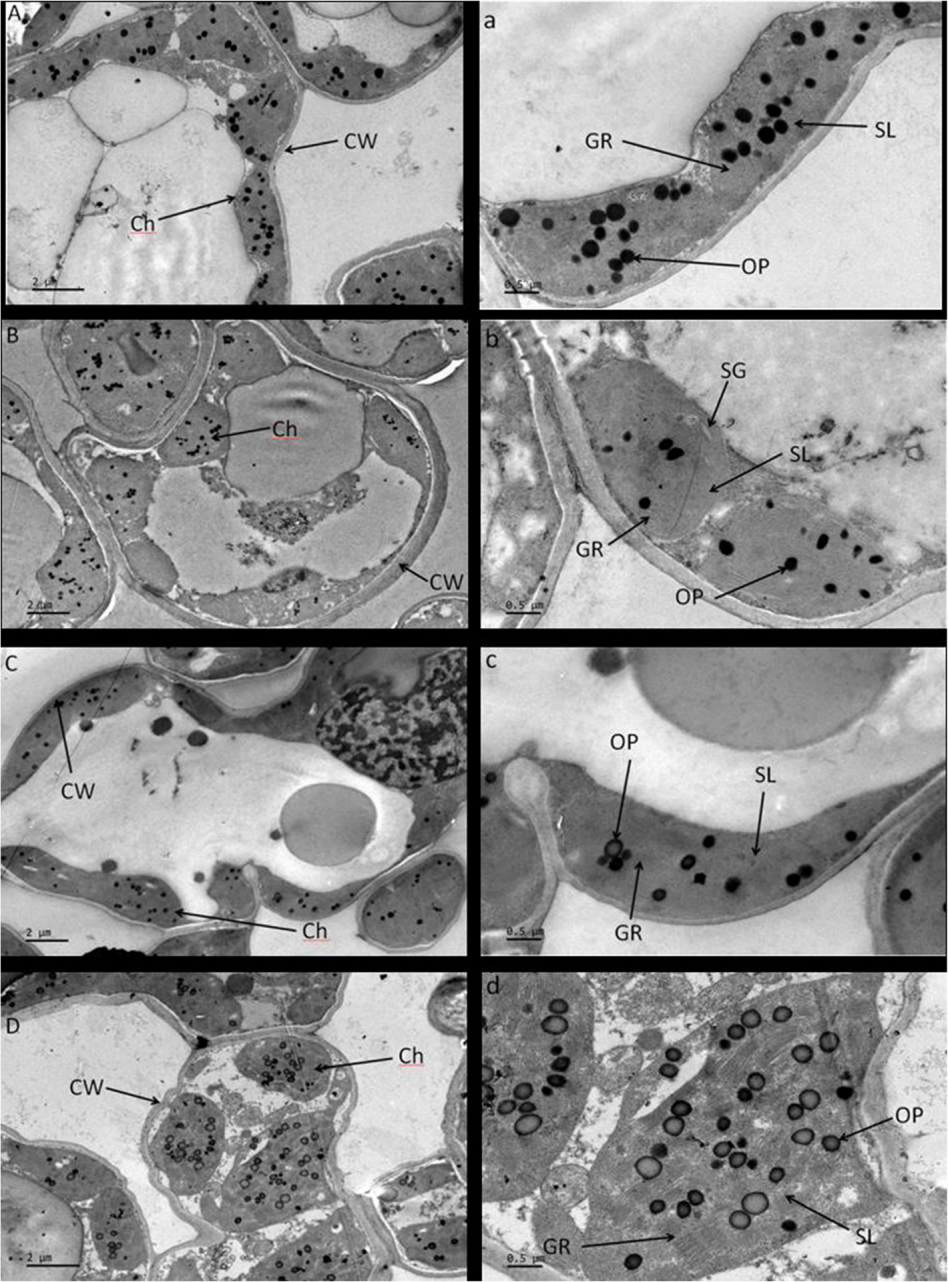
The ultrastructure of the leaf cell of *L. chinensis* in the different grazing intensity plots. (control, no grazing; LG, light grazing; MG, medium grazing; HG, heavy grazing). **a** control, **b** LG, **c** MG, **d** HG. Abbreviations: CW, cell wall; SL, stroma lamellae; G, granum; SG, starch grain; P, plastoglobuli. The scale bars for the whole cell (top row) and the enlarged parts (bottom row) are 2 and 0.5 μm, respectively

The degree of membrane lipid peroxidation differed significantly among the grazing intensities (Fig. 5, *P* < 0.05). The MDA contents of the leaves were significantly higher in the grazed plots than in the control, but did not differ significantly among the grazed plots.

**Fig. 6:**
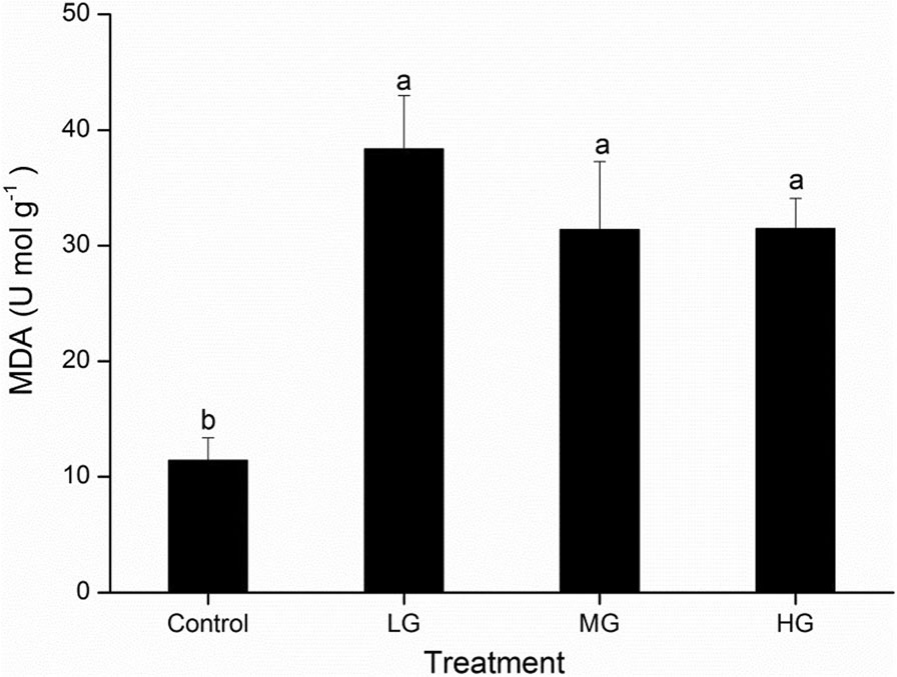
Leaf Malondialdehyde (MDA) content of *L. chinensis* in the different grazing intensity plots. (control, no grazing; LG, light grazing; MG, medium grazing; HG, heavy grazing). Values are means ± SE (*n* = 3). Values of a parameter labeled with different letters differ significantly between grazing intensities (ANOVA followed by LSD test, *P* < 0.05)

The leaves had significantly higher proline contents in the MG plots than in the other plots, which did not differ significantly (Fig. 6a). SOD activity was significantly lower in the HG plots than in the other plots, which did not differ significantly. POD activity was significantly lower in the LG plots than in the other plots, and significantly higher in the HG plots. CAT activity was significantly higher in the MG plots than in the control, but did not differ significantly among the other plots.

## Discussion

### Morphological changes in response to increasing grazing intensity

Morphological changes of plants are a primary response to environmental stresses. As the main organ consumed by herbivores, the leaves of *L. chinensis* were significantly influenced by herbivory. SLA is an important indicator of leaf function, as it represents the plant’s ability to acquire and utilize resources, as well as the plant’s allocation of resources among different uses [36]. In the LG plots, *L. chinensis* had the highest SLA, which means that *L. chinensis* can grow new leaves fastest after light herbivory and that its response to the uneven distribution of light resources became more flexible [25, 60]. Plants with high SLA can respond more easily to a resource-rich environment, such as the increased C, N contents in the soil or the availability of light after grazing. However, high SLA also indicates thinner cell walls, which makes the leaves more vulnerable to damage by herbivores [78]. In contrast, the low SLA in the MG plots suggested that the leaves became thicker than those in the control and probably lasted longer. Meanwhile, leaves accumulated more assimilate per unit area, which would offer some protection against herbivores [36, 44]. At the same time, the steadily decreasing plant height and internode length with increasing grazing intensity produced plants with more of a dwarf phenotype, which may represent a grazing avoidance strategy that protects the plants. On the other hand, the increase in tiller number with increasing grazing intensity would promote regrowth of the plants, and could also represent a grazing tolerance mechanism [65]. At the heavy grazing intensity, roots of *L. chinensis* absorbed more nutrients rapidly from the soil to grow complementally, which also reduced the soil nutrient in the heavy grazing plots [76]. With increasing grazing intensity, plant biomass gradually decreases [62]. Once the pressure from grazing was removed, the proportion of *L. chinensis* in the vegetation cover would increase again. However, while grazing continues, dwarfing of the grazed plants can directly decrease grassland productivity [88]. Different grazing intensities resulted in the different morphological responses. However, we did not find the obvious morphological compensatory growth.

### Photosynthetic responses and adaptation to increasing grazing intensity

In addition to the abovementioned morphological changes, *L. chinensis* will adapt its physiology in response to the disturbance and to the resulting changes in resource availability [40, 70]. Grazing, herbivores will change light availability and thereby affect acquisition of this resource by plants, thereby affecting their physiological characteristics. Plant photosynthesis is a particularly important indicator of physiological sensitivity to environmental stress because this process provides the energy that plants need to survive and adapt [7]. In the present study, the photosynthetic rates of *L. chinensis* in the LG and MG plots were significantly lower than in the control, but increased in the HG plots. This suggests that the plants performed compensatory photosynthesis in response to the severe loss of leaves in the HG plots [15, 42]. In the LG plots, *L. chinensis* had a high LSP and low LCP, thereby allowing the plants to maximize their utilization of the available light energy. On the other hand, the plants had lower quantities of the photosynthetic pigments. The changes in the amounts and composition of the photosynthetic pigments reflect underlying functional modifications of the photosynthetic apparatus, thereby causing photosynthetic performance to change in a coordinated manner [17, 21]. Photosynthetic pigments are sensitive to environmental changes, making them potentially suitable as biomarkers [48]. Our results suggest that light grazing may inhibit chlorophyll synthesis or increase the activity of chlorophyll-degrading enzymes [61]. Through the chlorophyll cycle, Chl *b* can be converted into Chl *a*. This interconversion gives plants the ability to optimally adapt to changing light conditions [61]. There was no significant difference in Chl *a*/*b* among grazing plots. As Chl *b* is only present in the light-harvesting phase and not the light energy transformation phase, the lower Chl *a*/*b* suggests that they invested more in the absorption of light energy. If the photosystems had significantly larger antenna sizes (a significantly lower Chl *a*/*b* ratio), this could represent more optimal use of the low light intensity that would exist when there was little reduction of the vegetation cover [18, 73].

On the other hand, more absorbed light energy cannot always be fully used to support photosynthesis, and the excess energy must be dissipated. The increased thermal energy dissipation in all grazed plots was accompanied by increased *F*_o_, which may indicate the degradation of the D1 protein in PSII or disruption of the energy transfer into the reaction center [31]. Under stress, *F*_o_ would increase but *F*_v_/*F*_m_ would decrease [51] Two factors may lead to this situation: a reduction of the plastoquinone electron receptors or incomplete oxidation, which delays the electron transfer chain in PSII; the damage to the light-harvesting phyllochlorin [5]. Therefore, plants had lower ETR and higher ROS accumulation (as measured by the MDA content) in the LG plots. The increase of *F*_o_, together with the decreased ETR and *F*_v_/*F*_m_ in the LG plots indicated damage to the photosynthetic apparatus, which would also explain the decreased Rubisco activity [41]. In addition, there is often a correlation between *F*_o_ and the Chl contents in the plants: *F*_o_ increases with decreasing Chl content. We observed starch grains in the chloroplasts in the LG plots. This may due to damage to the photosynthetic apparatus. The photosynthesis-synthesized sugars cannot be exported or reduced in time and are therefore converted into starch, resulting in the accumulation of starch granules in the chloroplast.

The Chl *b* content did not differ significantly among the four plots, suggesting that this pigment was not sensitive to grazing stress. However, with increasing grazing intensity, the Chl *a* content increased, which suggests that the plants responded to the more serious grazing stress by increasing the amount of the photosystem components used to improve photosynthetic efficiency by improving electron transfer among the reaction centers [21, 55]. In the MG and HG plots, the Car contents were higher than in the LG plot, which represents a protective mechanism for the photosystem. Car reacts with lipid peroxidation products and removes singlet oxygen [57]. The high Car content in the two most severely grazed plots would promote energy transfer from the chlorophyll molecules to a chlorophyll zeaxanthin heterodimer, thereby assisting with the dissipation of excess energy [32, 58]. Therefore, ROS accumulation decreased in the two plots, although the decrease was not significant. Especially in the MG plots, more energy was used for thermal energy dissipation (62%) and less was used for photosynthetic electron transport (19%). The higher NPQ in these plots suggests that that photosynthetic apparatus dissipated the excessive energy as heat and protected against photoinhibition [59, 64]. Φ_PSII_ represents the quantum efficiency of photochemical reactions and is negatively correlated with NPQ [72]. Therefore, with lower Φ_PSII_ in the MG plots, the photosynthetic rate decreased compared to the other plots. This also explains why the C accumulation in the leaves was lower in these plots.

Our results suggest that compensatory photosynthesis occurred in the HG plots. More energy was used for the photosynthetic electron transport (27%) than in the other plots (19 to 25%), thereby increasing the photosynthetic rate compared with the other grazed plots. With increasing grazing intensity, the transpiration rate of leaves increases. Plants in the HG plots therefore increased their WUE compared to the other grazing plots to maintain growth. Furthermore, plants absorbed more nutrient elements form the soil, which affected the photosynthesis [1]. The leaf N contents were highest in the HG plots. The significantly increased *J*_max_/*V*_cmax_ ratio in the HG plots also suggested that the plants invested more N in Rubisco carboxylation [47]. The high N content, photosynthetic pigment, Rubisco activity, and more energy in the photosynthetic electron transport all indicated the compensatory photosynthesis of plants in the HG plots [19]. Though the chloroplasts were clearly damaged in the HG plots, adjustment of the plant’s internal mechanisms improved the compensatory photosynthesis. A certain degree of herbivore stimulated the increase of photosynthetic rate. In contrast, the increased leaf P contents in the LG plots suggest increased sensitivity of *V*_cmax_ to the leaf N content [79]. We hypothesize that there was a trade-off between the photosynthetic gains and energy dissipation costs to improve survival of the plants. In other hand, the adjustment of photosynthesis in the different intensity grazing plots was also influenced by the change of community. In general, the leaf area index (LAI) decreased significantly with the drop of above-ground biomass. That’s one of the reasons the photosynthetic capacity decreased in the grazing plots. However, the relationship between the photosynthetic adjustment and community change in the different grazing plots still need to be further discussed with data support. Besides, the compensatory photosynthesis cannot be confined to the defoliation simply. The other environmental factors including the nutrients and water also should be taken into account [3].

### ROS regulation under different grazing intensities

Under grazing, excess light will contribute to the production of ROS, which directly damage the cell and chloroplast membranes [38]. Under these circumstances, malondialdehyde is produced, so the malondialdehyde level provides a good proxy for oxidative damage [48, 52]. In this study, the MDA contents of the leaves in the grazing plots were significantly higher than those in the control plots, which suggest that grazing stress resulted in increased lipid peroxidation in the cell membrane. Furthermore, as the main organ affected by herbivores, leaves have higher MDA contents, thereby increasing the contents of free fatty acids and free sterols and decreasing the fluidity of the cell membrane [50]. Especially in the LG plots, lipid peroxidation was serious and would have led to photodamage of PSII in the chloroplasts [47]. As we noted earlier in the Discussion, the high Car content could quench the trilinear chlorophyll before it reacts with oxygen, and could also quench singlet oxygen [58]. Therefore, a high Car content could have helped to decrease the ROS content in the MG and HG plots.

Once the ROS content increases to a certain level, the antioxidant system will be activated to scavenge the ROS [4, 35, 38]. SOD is the first line of defense against ROS and will convert superoxide radicals into H_2_O_2_ [52]. Then peroxidase and catalase convert H_2_O_2_ into harmless H_2_O_2_ and O_2_. In this study, the activity of superoxide dismutase was similarly high in the control, LG, and MG plots, but significantly lower in the HG plots, suggesting that the highest level of grazing stress suppressed the activity of this enzyme because the higher *P*_n_ used up more light energy and therefore reduced production of ROS compared to the other grazing levels. However, high activity of SOD alone may not have been sufficient to increase tolerance of grazing stress and decrease membrane lipid peroxidation damage in the LG plots [2]. The MG plots had the highest catalase activity. In contrast, when the SOD and CAT activity decreased, POD activity increased significantly in the HG plots. The significant increase in both POD and CAT enzymes with increasing grazing intensity suggests that superoxide dismutase converted much of the ROS into H_2_O_2_, which these enzymes then converted into O_2_.

The balance among the activity of different antioxidant enzymes is crucial for decreasing the ROS level [4, 11]. In addition, the increased water stress in the grazing plots (indicated by significant decreases in the leaf osmotic potential at the two highest grazing intensities) would have induced the generation of ROS. However, the production of these antioxidant enzymes was not adequate to mitigate the negative influence of the ROS. Therefore, the accumulation of proline in the MG plots would have increased the osmotic potential to protect the cell [38, 54]. In the present study, the low leaf osmotic potential in the MG plots would transmit a signal that would accelerate the production of proline. The proline increase in MG plots could compensate for this decrease of the antioxidant enzymes [33].

These results show how the grazed plants adapted a combination of various strategies to mitigate the effects of ROS generation or to scavenge excess ROS and counteract the grazing stress.

## Conclusions

We found that under grazing disturbance, *L. chinensis* adopted a range of strategies to growth. When the grazing pressure was light to medium, *L. chinensis* exhibited decreased photosynthetic capacity. More light was absorbed, but the excess energy could not be dissipated sufficiently fast, leading to damage to the photosynthetic apparatus and accumulation of ROS, as indicated by the high level of MDA. The accumulation of ROS also induced increased POD and CAT activity to protect the cell. A new equilibrium would therefore develop. Though the photosynthetic capacity decreased, the plant’s productivity was not significantly decreased by light grazing. As the grazing intensity increased, *L. chinensis* began to reduce the production of ROS by increased thermal energy dissipation. The more energy that was dissipated, the less energy was used to support photosynthetic electron transport. At the same time, the increased herbivore intensity induced further declines in productivity. Under the heaviest grazing pressure, plant productivity (aboveground biomass) decreased sharply. Plants therefore changed their strategy to increase survival through photosynthetic compensation.

Our results suggest that the photosynthetic mechanisms of *L. chinensis* were able to adjust so as to acclimate to different levels of grazing intensity. The adjustments of the photosynthetic enzymes, pigments, chlorophyll fluorescence parameters, and contents of antioxidant enzymes all contributed to this acclimation. The degree of photoinhibition depended on the balance between the plant’s photosynthetic protection mechanisms and the damage to PSII. However, the relationship between photosynthetic performance and total productivity was not always consistent. More research will therefore be necessary to clarify this relationship.

## Methods

### Study site and experimental design

**Fig. 7:**
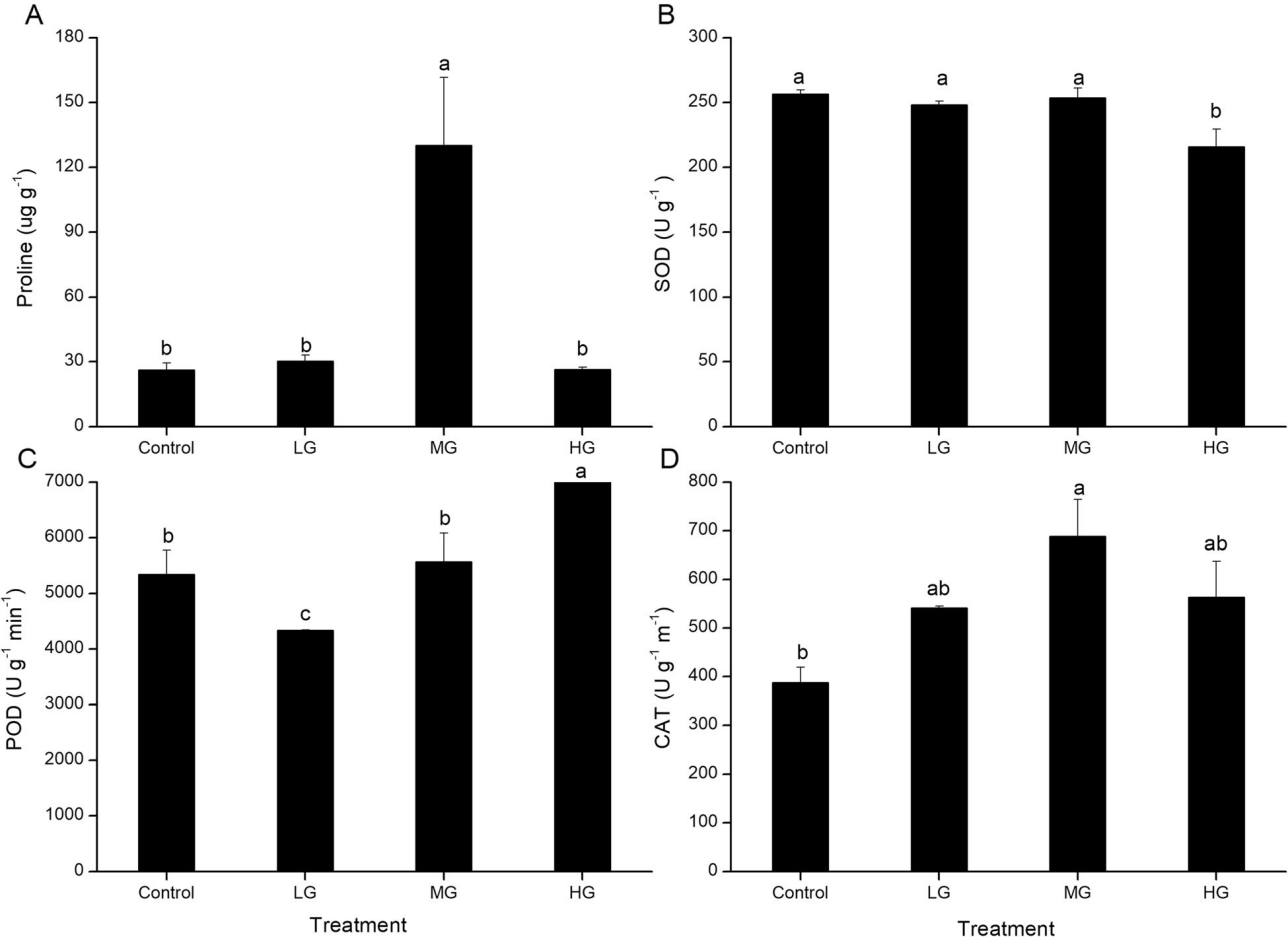
The proline content, SOD, POD, and CAT activity of *L. chinensis* in the plots with different grazing intensities (control, no grazing; LG, light grazing; MG, medium grazing; HG, heavy grazing). Values are means ± SE (*n* = 3). Note that the y-axis scales differ greatly among the graphs. Values of a parameter labeled with different letters differ significantly among grazing intensities (ANOVA followed by LSD test, *P* < 0.05)

The study was conducted at the Grassland Ecosystem Research Station of the Chinese Academy of Sciences (116°42′E, 43°38′N to 44°49′N) in Xilinhot, Inner Mongolia, China. This region has a typical temperate continental monsoon climate, with annual precipitation of 400 mm (of which 70% falls from June to August) and a mean annual temperature of 0.7 °C. The temperature ranges from a minimum of − 41.1 °C in January to a maximum of 38.5 °C in August. The dominant species include *L. chinensis*, *Stipa grandis*, and *Stipa krylovii*. In June 2014, we began our grazing experiment, which lasted for 3 months. We designed four grazing intensity treatments: (1) a control, with no grazing; (2) light grazing (LG), with 4 sheep in each plot; (3) medium grazing (MG), with 8 sheep in each plot; and (4) heavy grazing (HG), with 12 sheep in each plot. Each plot was fenced to prevent changes in the number of sheep. Three replicates of each treatment were randomly assigned to 12 permanent plots, and each plot covered an area of 1.37 ha (Fig. 7). Each plot was about 125 m long and 110 m wide. The middle road was 5 cm wide. We chose *Leymus chinensis* (Trin.) TZvel., as the experimental subject in the 2016 growing season. We have permissions to collect such samples. The voucher specimen of *L. chinensis* was deposited in the Hulunbuir grassland ecosystem national field science research station of Inner Mongolia and the deposited number was 09–6045. The permission was obtained from the Grassland Ecosystem Research Station of the Chinese Academy of Sciences.

### Plants morphological traits biomass, and soil traits

During mid-August 2016, we established three quadrats (1 m × 1 m) within each grazing plot. We then randomly selected 10 individuals of *L. chinensis* in each replicate to measure the plant height, tiller number, and internode length. The leaf fresh area, fresh mass, and oven-dry mass were measured to determine the specific leaf area (SLA), which equaled the fresh leaf area divided by the leaf oven-dry mass. We also measured the leaf osmotic potential using a model WP4C Dewpoint PotentiaMeter (Decagon Devices, Pullman, WA, USA).

On the same date, we harvested the total aboveground biomass of *L. chinensis* to a height of 2 cm above the ground in the quadrats. The samples were then oven-dried at 65 °C to constant weight to determine the biomass. In addition, we ground the dried samples to a powder and then analyzed the powder to determine the leaf C, N, and P contents. The leaf C contents were determined by the Walkley-Black wet oxidation technique [56] with an automatic elemental analyzer (Vario EL, Elementar, Langenselbold, Germany). The leaf N contents were measured by the Kjeldahl method [9] with an automated Kjeldahl analyzer (Kjeltec 8400, Foss, Denmark). The leaf P contents were measured by inductively coupled plasma-atomic emission spectroscopy (ICP-AES) according to the method of Soon and Kalra [69]. In short, each sample and standard were weighted and mixed with nitric acid and H_2_O_2_. Then they were placed in the Teflon tank and tightened by the stainless steel sleeve. Then the samples were heated at 160 °C for 4 h. After cooling, the samples were measured by the ICP-AES.

At the same time, we collected the 10–20 cm soil with plastic packaging bags. The soil C, N, and P contents were measured by the same methods. The fresh soil was weight and then oven-dried at 105 °C to measure the dry mass. The soil water content was calculated by the following formulas:

$$ \mathrm{Soil}\ \mathrm{water}\ \mathrm{content}=\left(\mathrm{fresh}\ \mathrm{mass}-\mathrm{dry}\ \mathrm{mass}\right)/\mathrm{fresh}\ \mathrm{mass} $$

### Leaf gas-exchange and chlorophyll fluorescence parameters

Leaf gas-exchange and chlorophyll fluorescence parameters were measured by a portable gas-exchange system (LI-6400; Li-Cor, Lincoln, NE, USA). We used the fluorescent chambers (LI-6400-40) with the red and blue light source, which including 90% red light and 10% blue light. For these measurements, we chose 10 individuals of *L. chinensis* in each replicate of the four plots. For selecting plant samples, we chose the third leaf of each *L. chinensis* plant for photosynthetic measurements. The leaves were mature and healthy. We measured the instantaneous net photosynthetic rate (*P*_n_) and transpiration rate (*T*_r_), and then calculated the water-use efficiency (WUE) as *P*_n_/ *T*_r_. We determined the light-response curves of *L. chinensis* at a CO_2_ concentration of 380 μmol mol^− 1^ and a photosynthetically active radiation ranging from 0 to 2500 μmol m^− 2^ s^− 1^. The photosynthetic photon flux density (*PPFD*) was set at 2500, 2000, 1500, 1000, 500, 200, 150, 100, 50, 20, and 0 μmol m^− 2^ s^− 1^.The light-saturation point (LSP), light-compensation point (LCP), dark respiration rate (*R*_d_), and apparent quantum yield (AQY) were obtained from the light response curve. The calculation was based on the corrected nonrectangular hyperbolic model [74].

The CO_2_ response curves were measured with the CO_2_ concentration ranging from 0 to 1800 μmol mol^− 1^. The CO_2_ concentration was set at 400, 300, 200, 150, 100, 50, 400, 400, 600, 800, 1000, 1200, 1500, and 1800 μmol mol^− 1^. We calculated the maximum electron transport rate (*J*_max_), the maximum carboxylation efficiency (*V*_cmax_), and the triose phosphate utilization rate (*V*_TPU_) using the biochemical model of photosynthetic CO_2_ assimilation [23].

The plants were measured in a randomized order between 09:00 and 11:00 am. To make the conditions as consistent as possible, we set the same leaf external environment. The flow rate was set as 500 μmol s^− 1^ and the leaf temperature was 28 °C.

Chlorophyll fluorescence parameters were also measured by the Li-Cor LI-6400. Before the measurements, leaves must be wrapped with tinfoil for dark adaptation at the ambient temperature. The dark adaptation lasted for at least 40 min. The maximum fluorescence (*F*_m_) and minimum fluorescence (*F*_0_) in the dark-adapted state were recorded simultaneously in the darkness. Next, the leaves were exposed to a *PPFD* of 1000 μmol photons m^− 2^ s^− 1^ for more than 10 min to measure the minimum fluorescence (*F*_o_’), the maximum fluorescence (*F*_m_′), and the steady–state fluorescence (*F*_s_) in the light-adapted state after *P*_n_ stabilized. We then calculated the maximum quantum efficiency of photosystem II (PSII; *F*_v_/*F*_m_), the energy harvesting efficiency of PSII (*F*_v_’/*F*_m_′), the photochemical quenching coefficient (*q*_P_), the non-photochemical quenching coefficient (NPQ), the electron transport rate (ETR), the effective quantum yield of PSII (Φ_PSII_), and the energy partitioning among photosynthetic electron transport energy (*P*), the thermal energy dissipation (*D*), and excess energy (*E*) according to the methods of Demmig-Adams et al. [14].

$$ {\displaystyle \begin{array}{c}P=\left({F}_{\mathrm{m}}\hbox{'}-{F}_{\mathrm{s}}\right)/{F}_{\mathrm{m}}\hbox{'}\\ {}D=1-\left({F}_v\hbox{'}/{F}_m\hbox{'}\right)\\ {}E=\left({F}_{\mathrm{v}}\hbox{'}/{F}_{\mathrm{m}}\hbox{'}\right)\times \left(1-{q}_{\mathrm{P}}\right)\end{array}} $$

#### Leaf photosynthetic pigments and Rubisco activity

After measuring of photosynthesis, we selected the plants and immediately collected the leaves. The fresh samples was weighted and packaged in 0.5 g by the brown paper. Then the samples were frozen in liquid nitrogen for subsequent biochemical analyses.

We extracted the chlorophyll a (Chl *a*), chlorophyll b (Chl *b*), and carotenoids (Car) from the leaves using 95% ethanol at 25 °C in the darkness. We measured the absorbance of the resulting solutions at 470, 649, and 665 nm, respectively, using the 756PC ultraviolet-visible spectrophotometer. We then calculated the pigment contents using the equations of Fargašová [22].

We measured the Rubisco activity in the leaves according to the method of Wang et al. [80]. In short, the frozen samples were ground in extraction solution that contained 0.1 mol/L Tris-HCL, 12 mmol/L MgCl_2_, 0.36 mmol/L EDTA, and 5 mmol/L β-mercaptoethanol. We then centrifuged the solution at 1500 *g* and 4 °C for 15 min. We mixed 0.5 mL of the supernatant with 1 mol/L Tris-HCl (pH = 8.0), 2 mmol/L NADH, 0.1 mol/L MgCl_2_, 50 mmol/L DTT, 2 mol/L KHCO_3_, 1 mmol/L EDTA, and 160 U/L 3-phosphoglyceric phosphokinase. We then measured the absorbance at 340 nm using the 756PC ultraviolet-visible spectrophotometer to calculate the Rubisco activity.

$$ \mathrm{the}\ \mathrm{Rubisco}\ \mathrm{activity}=\frac{\varDelta {\mathrm{OD}}_{340}\times {\mathrm{V}}_{\mathrm{t}}}{2\times T\times {\mathrm{V}}_{\mathrm{s}}\times \mathrm{W}\times \mathrm{6.22.}} $$

ΔOD_340_ was the change absorbance in the reaction time. Two means that 2 mol NADH was oxidized when 1 mol CO_2_ was fixed. *T* was the reaction time. V_t_ was the extracting solution volume. V_s_ was the volume of the reaction solution. W was the weight of the sample. Six point twenty-two was the extinction coefficient of 1 μmol NADH.

### The lipid peroxidation and antioxidant systems and proline contents

We used the malondialdehyde (MDA) content as a proxy for the level of lipid peroxidation. We measured the MDA content according to the thiobarbituric acid reaction [16]. The samples (0.5 g) were ground in 5% trichloroacetic acid (TCA) solution and then centrifuged at 1500 *g* for 10 min. The supernate was mixed with 0.67% thiobarbital acid (TBA) solution. The solution was boiled and centrifuged again. We measured the absorbance at 450 nm, 532 nm and 600 nm using the ultraviolet-visible spectrophotometer (756PC, Shanghai Jinghua, Shanghai, China). The MDA contents were calculated by the following formulas.

$$ {\displaystyle \begin{array}{c}{C}_{MDA}=6.45\left(O{D}_{532}-O{D}_{600}\right)-0.56\ O{D}_{450}\\ {}\mathrm{The}\ \mathrm{sample}\ \mathrm{MDA}\ \mathrm{content}=\frac{{\mathrm{C}}_{\mathrm{MDA}}\times {\mathrm{V}}_{\mathrm{t}}}{{\mathrm{V}}_{\mathrm{s}}\times \mathrm{W}}\end{array}} $$

C_MDA_ was the concentration of MDA in the reaction solution. V_t_ was the extracting solution volume. V_s_ was the volume of the reaction solution. W was the weight of the sample.

We measured the activities of the antioxidant enzymes (SOD, POD and CAT) according to the methods of Gong et al. [24]. The sample (0.5 g) was ground in liquid nitrogen and extracted in the sodium phosphate buffer (pH 7.0). The extracting solution was centrifuged at 15000 *g* for 20 min at 4 °C. The resulting supernatant was used for the enzyme assays. One unit of the SOD activity was defined as the amount enzyme required to inhibit the reduction of nitroblue tetrazolium (NBT) by half. The reaction solution contained 50 Mm sodium phosphate buffer (pH 7.8) with 100 μM Na_2_-EDTA, 130 mM methionine, 750 μM NBT, 20 μM riboflavin and 0.1 ml enzyme extract. Reactions were performed at 4000 lx irradiance by the fluorescent lamp for 20 min. Reaction in the dark was the blank control group. We measured the absorbance at 560 nm and calculated the enzyme activity.

$$ \mathrm{The}\ \mathrm{SOD}\ \mathrm{activity}=\frac{\left({\mathrm{OD}}_{\mathrm{CK}}-\mathrm{OD}\right)\times {\mathrm{V}}_{\mathrm{t}}}{{\mathrm{OD}}_{\mathrm{CK}}\times {\mathrm{V}}_{\mathrm{s}}\times \mathrm{W}\times 0.5} $$

OD_CK_ and OD were the absorbance of the blank control and samples, respectively. V_t_ was the extracting solution volume. V_s_ was the volume of the reaction solution. W was the weight of the sample.

When measuring the POD activity, the enzyme extract was mixed with the reaction solution which included 100 mM potassium phosphate buffer (pH 7.0), 20 mM guaiacol, and 20 mm^3^ H_2_O_2_. The absorbance value at 470 nm was immediately determined and recorded every 30 s for 4 min. POD activity was calculated according to the change of absorbance according to the formula.

$$ \mathrm{The}\ \mathrm{POD}\ \mathrm{activity}=\frac{\varDelta {\mathrm{OD}}_{470}\times {\mathrm{V}}_{\mathrm{t}}}{T\times {\mathrm{V}}_{\mathrm{s}}\times \mathrm{W}\times 0.01} $$

ΔOD_470_ was the change absorbance in the reaction time. *T* was the reaction time. V_t_ was the extracting solution volume. V_s_ was the volume of the reaction solution. W was the weight of the sample.

When measuring the CAT activity, the enzyme extract was mixed with 20 Mm H_2_O_2_ and the sodium phosphate buffer (PH 7.0). Then we measured the decrease in absorbance at 240 nm every 30 s for 4 min.

$$ \mathrm{The}\ \mathrm{CAT}\ \mathrm{activity}=\frac{\varDelta {\mathrm{OD}}_{240}\times {\mathrm{V}}_{\mathrm{t}}}{T\times {\mathrm{V}}_{\mathrm{s}}\times \mathrm{W}\times 0.1} $$

ΔOD_240_ was the change absorbance in the reaction time. *T* was the reaction time. V_t_ was the extracting solution volume. V_s_ was the volume of the reaction solution. W was the weight of the sample.

We determined the proline content by the ninhydrin coloration method with some modification [8]. The samples (0.5 g) were ground in 5 ml 3% sulfosalicylic acid to boiling and centrifugation. Then 2 ml supernatant was mixed with 2 ml distilled water, 2 ml glacial acetic acid and 4 ml ninhydrin and boiled at 100 °C for 60 min. After cooling, we added toluene and measured the absorbance at 520 nm. The standard curve of proline was obtained by the same way.

$$ \mathrm{The}\ \mathrm{sample}\ \mathrm{proline}\ \mathrm{content}=\frac{\mathrm{C}\times {\mathrm{V}}_{\mathrm{t}}}{{\mathrm{V}}_{\mathrm{s}}\times \mathrm{W}} $$

C was the content of proline from the standard curve. V_t_ was the extracting solution volume. V_s_ was the volume of the reaction solution. W was the weight of the sample.

### Ultrastructure of the leaf cells

We examined the ultrastructure of the leaf cells by means of transmission electron microscopy [39]. We collected fresh leaves from the four plots (control, LG, MG, HG), then cut them into small pieces (1 mm × 1 mm × 2 mm) and fixed them in 4% glutaraldehyde solution at 4 °C. After washing the samples five times with 0.1 mol/L phosphate buffer, the materials were fixed overnight in 1% osmic acid at 4 °C. The materials were then dehydrated using an acetone series of 30, 50, 70, 85, 95, and 100% v/v (for 10 min at each concentration, 2 times). The materials were then infiltrated with acetone and resin: at proportions of 3:1 v/v, 1:1 v/v, and 1:2 v/v, in that order, with each infiltration conducted for 3 h the proportion was 3:1; 1:1; 1:2 respectively for 3 h followed by infiltration with 100% resin for 12 h. After polymerization at 60 °C for 24 h, we created thin-sections using an ultramicrotome (Leica Microsystems, Bensheim, Germany) and double-stained the sections with uranyl acetate–lead citrate. We examined the samples using a model JEM1230 transmission electron microscope (JEOL, Tokyo, Japan).

#### Data and statistical analysis

Statistical tests were carried out using version 20.0 of SPSS (SPSS Inc., Chicago, IL, USA). We tested for significant differences among the four grazing intensities using one-way analysis of variance (ANOVA), with significance at *P* < 0.05. When the ANOVA result was significant, we used least-significant-difference (LSD) tests to identify differences in the morphological and physiological traits between pairs of treatments. Before using ANOVA, normality of the data was tested by Kolmogorov-Smirnov Test. All graphs were created using version 8.5 of the Origin software (https://www.originlab.com/).

## Data Availability

The datasets generated and/or analysed during the current study are not publicly available but are available from the corresponding author on reasonable request.

## Abbreviations

AQY: Apparent quantum yield
CAT: Catalase
Ch: Chlorophyll
CW: Cell wall
D: Thermal dissipation
*D*: Thermal energy dissipation
*E*: Excess energy
ETR: Electron transport rate
*F*_m_: Maximum fluorescence yield in the dark-adapted state
*F*_m_′: Maximum fluorescence yield in the light-adapted state
*F*_o_: Minimum fluorescence yield in the dark-adapted state
*F*_o_’: Minimum fluorescence yield in the light-adapted state
*F*_s_: Steady-state fluorescence
*F*_v_’: Variable fluorescence in the light-adapted state
GR: Granum
HG: Heavy grazing
*J*_max_: The maximum electron transport rate
LCP: Light-compensation point
LG: Light grazing
LSP: Light-saturation point
MDA: Malondialdehyde
MG: Medium grazing
NBT: Nitroblue tetrazolium
NPQ: Non-photochemical quenching coefficient
OP: Osmiophilic granule
*P*: Photosynthetic electron transport energy
*P*_n_: Net photosynthetic rate
POD: Peroxidase
*PPFD*: Photosynthetic photon flux density
*q*_P_: Photochemical quenching coefficient
*R*_d_: Dark respiration rate
ROS: Reactive oxygen species
Rubisco: Ribulose 1,5-bisphosphate carboxylase
SL: Stroma lamellae
SOD: Superoxide dismutase
TBA: Thiobarbital acid
TCA: Trichloroacetic acid
*T*_r_: Transpiration rate
*V*_cmax_: The maximum carboxylation efficiency
*V*_TPU_: The triose phosphate utilization rate
WUE: Water-use efficiency
Φ_PSII_: Effective quantum yield of PSII
